# Understanding Candidozyma (Candida) auris: genomic evolution, antifungal resistance and the growing challenges in global infection control

**DOI:** 10.1099/jmm.0.002135

**Published:** 2026-03-24

**Authors:** Karoline Kristina Kemmerich, Suélen Andreia Rossi, João Nóbrega de Almeida Júnior, Arnaldo Lopes Colombo, Lysangela Ronalte Alves

**Affiliations:** 1 Departamento de Medicina, Disciplina de Doenças Infecciosas, Escola Paulista de Medicina, Universidade Federal de São Paulo, São Paulo, SP, Brazil; 2 Antimicrobial Resistance Institute of São Paulo (ARIES)-UNIFESP, São Paulo, SP, Brazil; 3 Laboratório de Regulação da Expressão Gênica, Instituto Carlos Chagas, FIOCRUZ Paraná, Curitiba, Brazil

**Keywords:** biofilm formation, environmental persistence, hospital-acquired infections, multidrug-resistant fungi, nosocomial outbreaks, virulence factors

## Abstract

*Candida auris* (recently renamed *Candidozyma auris*) is an emerging multidrug-resistant fungal pathogen, first identified in Japan in 2009. *C. auris* exhibits remarkable persistence on human skin and inanimate surfaces, resistance to multiple antifungals, notably fluconazole, and biofilm formation, which hinders infection control and leads to hospital outbreaks with high mortality rates. Despite ongoing research, key aspects of its reservoir origin, transmission routes and the best way to combat its spread and multidrug resistance remain unclear. Improving genomic surveillance and antifungal strategies is crucial to contain its spread and mitigate the growing public health threat posed by this resilient and potentially fatal fungal pathogen.

## Graphical Abstract

Infographic highlighting *Candidozyma auris*: a globally emerging, multidrug-resistant fungus with six clades, high mortality, hospital persistence, antifungal resistance, biofilm formation, stress response and environmental adaptability.

## Historical perspective

### Emergence and expansion of *Candida auris*

The first report of *C. auris* was in Tokyo, Japan, in 2009, when it was isolated from the ear canal of a patient hospitalized at the Tokyo Metropolitan Geriatric Hospital who was diagnosed with otitis. Since then, *C. auris* quickly emerged as a pathogen of great concern to the medical and scientific communities, as little was known about this newly identified species. Notably, *C. auris* began to appear sporadically in nosocomial settings across multiple countries, displaying high levels of resistance to various antifungals and causing fungemia in critically ill patients [1,2].

This initial Japanese report characterized the species based on its genotypic features, particularly the D1/D2 and internal transcribed spacer (ITS) regions. These regions showed similarity to other human pathogens, such as *Candidozyma haemuli*, *Candidozyma duobushaemuli*, *Candidozyma pseudohaemuli* and *Candidozyma vulturna*, all belonging to the *Metschnikowiaceae* family [1,3].

*C. auris* is a heterotrophic, aerobic, haploid yeast, formed by spherical or oval blastoconidia, rarely producing hyphae or pseudohyphae. It ideally grows at 37 °C, but also exhibits growth at 40–42 °C, albeit slowly. On CHROMagar Candida^®^, it forms pink colonies, which may vary to purplish-white or reddish; on CHROMagar Candida^®^ Plus, the colonies are light blue with a blue halo [1,4, 5]. For *C. auris*, the diagnosis is confirmed by matrix-assisted laser desorption/ionization-time of flight/MS or molecular tools, such as ITS or genome sequencing [6].

Currently, *C. auris* has been reported in over 50 countries, with cases detected on every continent except Antarctica. This widespread geographic distribution has raised concerns among healthcare professionals, as its presence is associated with hospital outbreaks and mortality rates ranging from 29% to 60% (Table 1). It primarily affects critically ill patients undergoing multiple invasive procedures and with intense exposure to antimicrobials, including immunocompromised patients. *C. auris* can develop multidrug resistance (MDR) and may persist on human skin and inanimate surfaces for prolonged periods. It has also been demonstrated to resist various disinfectant products [7,9].

**Table 1.** Comparative overview of *C. auris* clades

Clade	Geographical origin	Outbreak potential	MDR
l	South Asia (India, Pakistan)	High; several hospital outbreaks reported	High resistance to Fluc and moderate to Ampho; rare resistance to echinocandins
lI	East Asia (Japan, South Korea)	Low; most episodes are sporadic and superficial infections	Low rate of MDR: moderate resistance to Fluc and rare resistance to Ampho and echinocandins
lII	Africa (South Africa)	High; several hospital outbreaks documented	Variable; high resistance to Fluc and less than 5% to Ampho and echinocandins
IV	South America (Venezuela, Colombia)	High; several healthcare-associated outbreaks	Moderate resistance to Fluc and Ampho and rare resistance to echinocandins
V	Iran	Limited data; isolated cases reported	Limited data available: moderate resistance to Fluc and rare resistance to other antifungals
Vl	Singapore and Bangladesh	Limited data; isolated cases reported	Limited data available: few isolates tested were resistance to Ampho and susceptible to other antifungals

Table was based on review [59].

Ampho, amphotericin B; Fluc, fluconazole.

Recent studies have highlighted the significant clinical and economic burden associated with *C. auris* invasive infections. In a cohort of 56 patients, the 30-day all-cause mortality rate reached 28.6%, and clinical cure was achieved in only 57% of cases. Recurrence occurred in 29% of patients at 30 days and 13% at 90 days, underscoring the challenge of achieving sustained eradication [10]. *C. auris* candidemia also leads to prolonged hospitalizations, with median stays of 46–68 days, reflecting complex clinical management and high resource utilization [11].

Beyond individual outcomes, invasive fungal diseases, including *C. auris* infections, impose a substantial economic impact. In the USA alone, fungal infections collectively account for an estimated $19.4 billion annually in healthcare and productivity losses, illustrating the broader economic stakes of these emerging pathogens [12].

Post-outbreak investigations have further revealed that *C. auris* transmission is closely linked to intensive care environments. In one UK Intensive Care Unit (ICU), 94% of patients with *C. auris* infection had prior ICU admission, and the risk of colonization increased with hospital stay duration. Notably, reusable temperature probes were identified as a key source of transmission [13].

Invasive medical devices, including long-term intravascular catheters, dialysis lines, parenteral nutrition systems and external drains, further exacerbate infection risk by breaching protective barriers and providing surfaces for fungal adherence. *C. auris* is capable of forming persistent biofilms on these materials, turning medical devices into potential reservoirs for reinfection. Understanding the molecular mechanisms underlying biofilm formation is therefore essential to guide the development of improved preventive and therapeutic strategies [14].

### Eco-epidemiology

Outbreaks of *C. auris* have been reported in healthcare settings, particularly among patients requiring intensive care support with invasive medical devices and intense dysbiosis [15,17]. As previously noted, *C. auris* can persist in hospital environments for over 28 days, even after disinfection procedures [18,20]. Due to its resilience, *C. auris* spreads easily in healthcare facilities, often via shared medical equipment such as digital thermometers, bed rails, vital sign monitors, intravenous infusion pumps and trays [20,21]. Once an at-risk patient is colonized, skin colonization may persist for several weeks or months, both during hospitalization and after discharge to nursing homes, especially in patients undergoing invasive medical procedures and using medical devices such as drains, tubes and catheters in general [22]. Studies demonstrate that microbiota dysbiosis plays a significant role in skin disorders, such as atopic dermatitis, indicating that individuals with these conditions exhibit greater diversity in fungal interactions [23,25]. Sequencing the skin microbiomes of nursing home residents revealed negative associations between *Staphylococcus* and *Corynebacterium* species and *C. auris*, and that colonization by *Malassezia* spp. may limit the invasion of *C. auris* [25,26].

The exact environmental reservoir of *C. auris* remains uncertain and requires further investigation. In 2021, a study identified a possible reservoir for *C. auris* along the coast of the Andaman Islands, India. The pathogen demonstrated the ability to survive in extreme conditions, including high temperatures and salinity, which may contribute to its persistence in hospital environments [17,27]. In 2022, *C. auris* was also detected in marine and estuarine waters along the coast of Colombia. These findings reinforce its similarity to *C. haemuli*, a species frequently isolated from marine environments [28].

In addition to the hospital environment, *C. auris* has been isolated from domestic animals and fruits. The first record in the animal setting occurred in 2021 from an oral swab collected from a shelter dog in the USA (Clade IV). In 2023, four new cases were identified in dogs in India: three with otitis and one with cutaneous colonization, all belonging to Clade IV [29,30].

*C. auris* was isolated only on the surfaces of eight apples stored in commercial establishments in India, a hyperendemic area for this fungal pathogen. In this case, the isolates belonged to Clade I and showed high genetic proximity to clinical and environmental isolates from the coast of India. Despite these findings, its role in human transmission remains unclear [31].

### Factors contributing to the spread beyond global warming

The rapid and unexpected emergence of *C. auris* has been linked to global warming. This hypothesis suggests that its adaptive evolution is not solely due to natural selection but has also been significantly influenced by human activities [28].

Human impact on global health has created imbalances in the interconnected systems of human, animal, plant and environmental health [32]. Beyond climate change, other factors may be driving *C. auris* adaptation, including:

#### Indiscriminate use of antimicrobials

The excessive use of antifungals and antibiotics in human medicine, livestock and agriculture has led to widespread contamination of surface and groundwater sources, thereby fostering microbial evolution, adaptation and antimicrobial resistance [27,33, 34].

#### Environmental pollution

The accumulation of plastic waste in aquatic environments may also contribute to *C. auris* evolution. Microplastics, present at alarming levels in oceans, often contain significant amounts of antimicrobials. These microplastic surfaces could serve as novel reservoirs for pathogenic fungi [20,35,37].

#### Animal reservoirs

Although *C. auris* has not yet been widely detected in non-human hosts, additional cases may emerge as surveillance expands [38,42]. Several hypotheses suggest that *C. auris*, originally an environmental fungus, may have acquired the capacity to infect intermediate hosts, possibly avian species. This theory is supported by its notable thermotolerance: bird body temperatures typically exceed 40 °C, higher than the human average of 37 °C. Adaptation to such elevated temperatures could have facilitated the fungus’s transition to a human-pathogenic lifestyle [43]. According to Casadevall *et al*. [43], fungi capable of growth at 40–42 °C can infect birds, and *C. auris* may have disseminated from rural to urban settings via avian hosts. Similar ecological associations have been observed in other fungal pathogens, including *Candida glabrata* [44] and *Cryptococcus neoformans*, the latter frequently isolated from pigeon excreta [45,47].

In addition, clinical strains of *C. auris* can enter and persist in the environment through inadequately managed clinical waste. Improper disposal practices enable long-term survival and potential transmission beyond healthcare facilities (reviewed in [48]). Wastewater effluents may also serve as dissemination routes, as human excreta can contaminate sewage systems, a pathway previously demonstrated for *Candida albicans*.

Despite these growing insights, the mechanisms underlying *C. auris* emergence and persistence remain poorly understood. However, this pathogen possesses multiple survival strategies, including *MDR* to antifungals through drug efflux, *virulence factors* such as biofilm formation, *production of adhesins and proteases* and *cell wall modifications* [7,49,51].

## Microbial characteristics: genotypic features

### Genome structure and organization

The genome of *C. auris* is relatively small, with an average size of ∼12.3 Mb, and it consists of 5–7 chromosomes, depending on the strain [52,53]. Genomic analyses have revealed several unique characteristics that differentiate *C. auris* from other *Candida* species. Unlike *C. albicans*, which is diploid, *C. auris* is predominantly haploid, although polyploid forms have been reported in some strains, particularly those with drug-resistance adaptations [54].

Genome sequencing has also identified a large number of transposable elements in *C. auris* that may contribute to genome plasticity and facilitate rapid adaptation to environmental stresses and antifungal agents [52]. Additionally, subtelomeric regions in *C. auris* are enriched with genes encoding cell wall proteins, adhesins and other virulence- and biofilm-associated factors [55]. This arrangement suggests that subtelomeric regions play a role in environmental adaptation and immune evasion, as observed in other pathogenic fungi.

### Genetic diversity and clades of *C. auris*

Genomic and taxonomic studies have revealed substantial genetic diversity within *C. auris*, leading to its classification into six major clades (I–VI) based on geographic origin: Clade I (South Asia), Clade II (East Asia), Clade III (South Africa), Clade IV (South America), Clade V (Iran) and Clade VI (Singapore) [10,53, 56,62]. Although originally region-specific, several clades are now reported across multiple countries, likely due to global mobility and healthcare-associated transmission (Table 1) [10,56, 57]. This remarkable genetic divergence, unusual for an emerging pathogen, suggests that *C. auris* may have evolved independently in different regions before its recognition as a human pathogen [43,53, 62].

The clades differ in geographic distribution, virulence, antifungal susceptibility and clinical relevance. Clade I, first described in South Asia, is the most prevalent and exhibits extensive MDR, with most isolates resistant to fluconazole and showing elevated MICs for amphotericin B and eventually echinocandins [58,59]. Clade II, originating from East Asia, demonstrates the greatest antifungal susceptibility and lower pathogenicity, with limited persistence on mammalian skin [56,63]. Clades III and IV (South African and South American, respectively) may also develop MDR – particularly to fluconazole and amphotericin B, but most isolates retain susceptibility to echinocandins and have a narrower global distribution compared with Clade I [13,14, 58, 59]. Clades V and VI, more recently identified in Iran (2018) and Singapore (2023), remain under-characterized and mostly restricted to the areas of the original reports.

A recent global analysis of 304 *C*. *auris* isolates from 19 countries – including clinical and environmental samples – found that 97% of Clade I isolates were resistant to fluconazole and 47% to amphotericin B. Clade III isolates occasionally showed reduced susceptibility to micafungin, whereas Clade II isolates remained largely sensitive to fluconazole, amphotericin B and micafungin [56].

Overall, ∼90% of *C. auris* isolates worldwide are fluconazole-resistant, and 30% show reduced susceptibility to amphotericin B [64,65]. Clade-specific resistance patterns are clinically significant; for instance, Clade I often exhibits resistance to multiple azoles, while Clade IV – considered intrinsically fluconazole-resistant – shows variable echinocandin MICs [64,66]. These patterns emphasize the importance of considering regional clade distribution when selecting antifungal therapies and developing infection control strategies [52,67].

### Mechanisms of antifungal resistance

One of the most challenging aspects of managing *C. auris* infections is their multidrug-resistant phenotype. Genomic analyses have provided valuable insights into the molecular mechanisms underlying this resistance. The *ERG11* gene, which encodes lanosterol 14α-demethylase involved in ergosterol biosynthesis, is frequently mutated in azole-resistant strains of *C. auris*, particularly in Clades I and III [54]. Mutations in *ERG11*, such as Y132F and K143R (Clades I and IV) and F126L (Clade III), are strongly associated with fluconazole resistance, suggesting clade-specific evolutionary adaptation [56]. Moreover, *C. auris* expresses multiple efflux transporters, including *CDR1 *and *MDR1*, that actively expel azole compounds, compounding resistance [68].

Recent evidence has further clarified the genetic contributions of *ERG11* and *MRR1A* mutations to fluconazole resistance. Barker *et al*. [69] demonstrated that in Clade III isolates, the *ERG11*^VF125AL^ mutation is the primary driver of resistance, while the *MRR1A*^N647T^ mutation confers only a modest effect through *MDR1* overexpression. Notably, these mutations do not affect susceptibility to long-chain triazoles, such as itraconazole, posaconazole or isavuconazole [69].

Studies on resistance to amphotericin B are scarce, and apparently, mutations in *SSK1* and *HOG1* may contribute to polyene resistance [70]. Additional reports describe *ERG3* gene deletion [71] and Slower Growth on Non-fermentable carbon sources (SNG1) mutations associated with amphotericin B resistance [72]. Regarding echinocandin resistance, *FKS1* mutations, including S639F, F635Y, F635L, R1354S, R1354H, D642Y and R1354Y, have been identified and are known to reduce drug-binding affinity, thereby diminishing treatment efficacy [73,74] (reviewed in [64]).

Finally, *in vitro* serial exposure studies have demonstrated the potential of *C. auris* isolates to evolve into multidrug-resistant strains through copy number variations and mutations in genes such as *ERG2*, *CIS2*, *ERG3*, *ERG11* and *MEC3*, leading to increased MICs against multiple antifungal classes [75]. Together, these findings emphasize the genetic plasticity of *C. auris* and the urgent need for genomic surveillance to monitor emerging resistance trends.

## Microbial characteristics: phenotypic features and virulence factors

### Aggregation

Several studies have demonstrated that fungal morphology directly affects the virulence and pathogenicity of these micro-organisms [76,77], and that it also plays an important role in evading the host’s immune system [78]. *C. auris* can present an aggregating morphology, characterized by the formation of cellular clusters where cells remain attached even after multiple washing steps due to enhanced cell–cell adhesion. Studies indicate that this aggregation may be mediated by specific adhesins, including members of the Agglutinin-Like Sequence (ALS) family, which facilitate cell-to-cell adhesion and biofilm formation [79,82]. This phenotype can also result from a defect in cell division, leading to the failure to release daughter cells after budding [80]. Interestingly, cell aggregation profiles differ between clades, with Clade III strains exhibiting more aggregation [83].

The presence of the aggregative phenotype has implications for virulence and host immune response. Mouse skin infection models demonstrate that aggregative *C. auris* strains induce distinct inflammatory responses compared with non-aggregative strains, suggesting that aggregation may modulate host–pathogen interactions [82]. Additionally, cellular aggregation can impact antifungal susceptibility. Cell clusters may limit drug penetration, conferring a survival advantage under adverse conditions [79].

### Biofilm formation and adhesion

Biofilm formation is an important virulence factor in *Candida* species. *C. auris* can grow under adverse conditions, and its ability to form biofilms likely plays a key role in its environmental persistence and resistance to certain disinfectants [50]. When comparing *C. auris* biofilms with those of other *Candida* species, authors have found limited extracellular matrix production, and biofilms are primarily composed of budding cells [7,84]. The transcription factor Ume6 in *C. auris* plays a role in morphogenesis. The Ume6-hyperactivated strain (UME6^HA^) increases adhesion to surfaces and forms biofilms with greater biomass, highlighting its influence on biofilm development [85].

Biofilm formation in *C. auris* is associated with phase- and antifungal-class-dependent resistance profiles [86]. During the intermediate and mature stages of biofilm formation, several genes encoding efflux pumps are upregulated, including ATP-binding cassette transporters and major facilitator superfamily transporters. Inhibition of these transporters significantly increases susceptibility to fluconazole, demonstrating their role in antifungal resistance [86]. Additionally, studies show that the *C. auris* biofilm matrix is rich in mannan–glucan polysaccharides. This structure plays a crucial role in antifungal tolerance by sequestering nearly 70% of the available antifungal agents. This sequestration mechanism appears to be conserved among *Candida* species and contributes to biofilm-associated drug resistance [87].

*C. auris* biofilm profile has been evaluated in a synthetic sweat system and an *ex vivo* porcine skin model. The results show that *C. auris* forms biofilms in the synthetic sweat and skin models more efficiently than *C. albicans*. These findings provide insights into how *C. auris* strains efficiently colonize the skin, unlike other *Candida* species. This characteristic may contribute to the persistence of this fungus in hospital environments [88], as this pathogen has demonstrated efficiency in colonization and biofilm formation, as shown in the study.

The ability of *C. auris* to form biofilms on the skin is concerning, as this phenotype can directly affect the efficacy of chlorhexidine gluconate solutions. A 2% chlorhexidine gluconate solution is commonly used to cleanse the skin of hospitalized, bedridden patients. However, despite its *in vitro* activity, *C. auris* persists in patients bathed with this solution [4].

*C. auris* also possesses a large number of adhesins. The surface colonization factor 1 (Scf1), a *C. auris*-specific adhesin, and the conserved adhesin Iff4109 play important roles in the adhesion and association of the fungus with abiotic surfaces. Scf1 is necessary and sufficient for the robust attachment of *C. auris* cells to polymer substrates [89]. In addition, the adhesins Als5 and Scf1 are also essential for adhesion and biofilm formation in *C. auris* [90]. The protein Hog1 mitogen-activated protein kinase plays an important role in maintaining cell wall architecture, which is important for cell adhesion and colonization [70]. It is assumed that skin microbiota can influence the adhesion and colonization of *C. auris* on the skin [50]. Although information about the interaction between *C. auris* and other micro-organisms during skin colonization is limited, some studies suggest that patients with *Malassezia* species on their skin have a lower risk of *C. auris* colonization [26].

The fungal cell wall is the main recognition structure for the host immune system during host–pathogen interactions. This recognition occurs through pathogen-associated molecular patterns present in the fungal cell wall and pattern recognition receptors present on the surface of immune cells. Changes in the composition of the fungal cell wall can aid in pathogen evasion, as described in strategies already described in *Candida* spp. [91,92]. For example, one of the main strategies described in * C. albicans* is the masking of the β−1,3 glucan layer with mannoproteins, thus preventing pathogen recognition by immune cells. In *C. auris*, a study demonstrated that lactate-induced change in β-glucan reduced phagocytosis by a human acute monocytic leukemia cell line widely used as a model for monocyte/macrophage differentiation (THP-1) and a murine macrophage-like cell line commonly used for studies of inflammation and innate immune responses (RAW 264.7) cells.

### Proteases

Aspartic proteases (Saps) are enzymes that contribute significantly to fungal virulence and have been described in several *Candida* species, including *C. auris* [93,96], playing important roles in tissue damage and invasion, in the metabolic activity of biofilm cells and in the modulation of the host immune response [50,97,99]. It is important to emphasize that there may be differences in protease activity between the distinct clades of *C. auris* and according to the conditions to which the fungal cells are exposed [95].

### Environmental cleaning and disinfection

As previously mentioned, *C. auris* is a resilient pathogen with high environmental persistence, making infection control in hospital settings particularly challenging [100,103]. In a controlled environment mimicking the temperature and relative humidity conditions of healthcare environments, *C. auris* can survive and persist for up to 28 days on plastic surfaces [20].

Photodynamic therapy has been explored for its antifungal efficacy, particularly against fungal biofilms, as certain wavelengths can penetrate the protective biofilm layer, enhancing its effectiveness [104]. Assessment of the germicidal efficacy of ultraviolet radiation (100–280 nm) (UV-C) light (254 nm) against *C. auris* colonies revealed that 10 min of exposure did not significantly reduce growth. In contrast, prolonged exposure times of 20–30 min can substantially decrease colony growth.

Given the emergence of *C. auris*, the efficacy of various disinfectants, including alcohol, peracetic acid, acetic acid, phenolic compounds and glutaraldehyde, has been evaluated against this pathogen [103]. Hydrogen peroxide, alone or in conjunction with a chlorine-based product (1,000 p.p.m.), is active against dehydrated *C. auris* cells [105] and helped control a *C. auris* outbreak in a hospital in the UK [106]. Chlorine-based disinfectants, in the forms of sodium hypochlorite and sodium dichloroisocyanurate, are effective against multidrug-resistant micro-organisms and other *Candida* species [103].

Products that are effective against other species will not necessarily be effective against *C. auris*. Notably, disinfectants that rely solely on quaternary ammonium compounds exhibit relatively low efficacy against several pathogenic *Candida* species, including *C. auris* [107,108]. However, a study using a quaternary ammonia-based product in combination with alcohol (0.1% quaternary with 58% ethane) improved results against *C. albicans* and *C. auris*, demonstrating that quaternary ammonia, when combined with other products, can enhance their efficacy [109]. Glutaraldehyde at 2.4% showed activity after 1 min of contact, reducing the growth of *C. auris* and *C. albicans* [109].

In addition, an alcohol-based product containing 12% ethanol and 17.5% propan-1-ol showed activity, reducing colonization by *C. auris* [110]. Clique ou toque aqui para inserir o texto. Peracetic acid (3,500 p.p.m.) and two chlorine-based products (1,000 p.p.m. chlorine) also reduced the viability and delayed the formation of *C. auris* dry-surface biofilm [18].

As a strategy to contain the spread of *C. auris* in healthcare settings, a study evaluated the activity of bismuth nanoparticles against the planktonic form, including strains from different clades and biofilm formation. Through scanning electron microscopy, the authors reported that the bismuth nanoparticles directly impacted the morphology of the yeast and the structure of the biofilm. Overall, the results showed that the nanoparticles were effective against the planktonic form but were only moderately active in the setting of biofilm growth [111].

## Open questions

1. What are the precise environmental reservoirs and ecological niches of *C. auris*, and how do these contribute to its global spread?
2. How does *C. auris* evade the host immune system, and what are the key virulence factors involved in its pathogenicity?
3. What roles do global warming and environmental pollution play in the emergence and adaptation of *C. auris*?
4. How can genomic surveillance and antifungal strategies be improved to contain the spread of *C. auris* better?
5. Are there any animal reservoirs, and what is their role in terms of enabling sustained transmission of *C. auris* in the community?

## Abbreviations

ITS: internal transcribed spacer
MDR: multidrug resistance
Saps: aspartic proteases
Scf1: surface colonization factor 1
UME6^HA^: Ume6-hyperactivated strain
